# Magnetite Nanoparticles Encapsulated with PBS-PEG for AMF Hyperthermia

**DOI:** 10.3390/ma18020258

**Published:** 2025-01-09

**Authors:** Carlos Augusto Zanoni Souto, Fernando Gomes de Souza Junior, André Romero da Silva, Kaushik Pal

**Affiliations:** 1 Instituto de Macromoléculas Professora Eloisa Mano, Centro de Tecnologia-Cidade Universitária, Universidade Federal do Rio de Janeiro, Rio de Janeiro 21941-909, Brazil; 2 Programa de Engenharia da Nanotecnologia, COPPE, Centro de Tecnologia-Cidade Universitária, Universidade Federal do Rio de Janeiro, Rio de Janeiro 21941-909, Brazil; 3 College of Engineering and Computing, Florida International University, 10555 West Flagler St. EC3900, Miami, FL 33174, USA; 4 Departamento de Química, Federal Institute of Espírito Santo, Campus Aracruz, Avenida Morobá, 248, Aracruz 29192-733, Brazil; 5 University Centre for Research and Development (UCRD), Department of Physics, Chandigarh University, Mohali 140413, Punjab, India

**Keywords:** cancer, hyperthermia, polymer, magnetite, nanoparticulate carriers

## Abstract

Novel studies on typical synthesized magnetite nanoparticles were encapsulated into a poly (butylene succinate)/poly (ethylene glycol) copolymer (PBS-PEG). PBS was chosen because of its biocompatibility characteristics necessary for biomedical applications. PEG, as part of the macromolecular structure, increases the hybrid system’s solubility in an aqueous environment, increasing the circulation time of the material in the bloodstream. The immune system has difficulty recognizing particles with good solubility in an aqueous medium and with a diameter until 200 nm, preventing the body from eliminating the nanoparticles before the magnetic hyperthermia is performed. All the prepared materials were characterized by Fourier transform infrared spectroscopy (FTIR), scanning electron microscopy (SEM), energy dispersive spectroscopy (EDS), X-ray powder diffraction (XRD), thermogravimetric analysis (TGA), differential scanning calorimetry (DSC), dynamic light scattering (DLS), and zeta potential. The detailed investigated result executes the formulation developed in this work, showing it has potential and that further studies and analyses can be carried out so that the formulation can be improved, thus obtaining even better results.

## 1. Introduction

It is unquestionable that cancer poses a risk to public health, especially among developing countries [1]. Cancer ranks as the number one reason for death and is a crucial barrier to increasing life expectancy in every country around the world. Keeping with estimates from the World Health Organization (WHO) in 2019, cancer is the primary or secondary leading explanation for death before the age of 70 in 112 of 183 countries and ranks third or fourth in more than 23 countries. Cancer’s rising prominence as the number one explanation for death partly reflects marked declines in mortality rates of stroke and coronary cardiopathy conditions in many countries, if compared to cancer [2]. Investors around the world are on this subject, and the global cancer therapy market is estimated to be equal to USD 581.25 billion in 2030 [3].

Although new therapeutic methods have been discovered, there are still types of neoplasms for which medicine has no defined treatment strategy. Thus, a technique called magneto hyperthermia has received particular attention since magnetic nanoparticles are directed to the tissue of interest, where they generate heat through an alternating magnetic field. Hyperthermia (HT) is a type of cancer treatment in which body tissue is exposed to high temperatures (up to 45 °C). By damaging proteins and internal cell structures, hyperthermia shrinks tumors [4,5]. However, the successful application of this method relies heavily on the effective delivery and stability of the nanoparticles within the biological environment. Magnetic nanoparticles are responsible for the generation of HT [6]. Alternating magnetic fields (AMFs) magnetize and demagnetize these materials in a random direction, quickly increasing the temperature [7].

Under AMFs, the iron oxide nanoparticles, besides generating heat, have promising clinical applications due to their biocompatibility and small diameters [8]. They can also be modified with the appropriate coatings, including organic molecules like polymers, dextran, dendrimers, and chitosan. Moreover, modifications can occur with inorganic molecules, like gold, to forestall aggregation and increase their circulatory half-life [9].

On the other hand, magnetic iron oxide nanoparticles present small surface zeta potential values, producing a solid tendency to agglomerate. For clinical use, the particles must be covered with a biocompatible material to take care of colloidal stability and magnetic properties. Polymers are the most widely used materials for preparing magnetic nanocomposites, which are more interesting than pure NPMs for biomedical applications [10].

Among polymers, poly (butylene succinate) (PBS) is obtained from renewable sources by the polycondensation reaction between succinic acid and 1,4-butanediol. Fermentative routes can produce both reagents, featuring a new biotechnological and sustainable route for the production of polymers [11,12,13].

In turn, polyethylene glycol (PEG) was chosen to increase the hydrophilicity of nanoparticles. PEG is a linear polyether diol, commonly used in a wide range of human intravenous formulations, being considered immunologically safe as it is eliminated from the intact body by the kidneys [14,15]. The high solubility of PEG in water inside the body produces an effect called biomasking, preventing the immune system from recognizing the nanoparticles as foreign bodies, thus increasing their circulation time [16]. This way, the medication will be biocompatible and will stay in the body long enough for the hyperthermia sections.

This study aims to address these challenges by synthesizing magnetite nanoparticles encapsulated in a poly (butylene succinate)/poly (ethylene glycol) (PBS-PEG) copolymer. The choice of PBS is motivated by its biocompatibility, making it suitable for biomedical applications. At the same time, PEG enhances the solubility of the hybrid system in aqueous environments, prolonging circulation time in the bloodstream. By developing a nanocomposite that combines the advantages of magnetic hyperthermia with improved biocompatibility and stability, this research seeks to significantly advance targeted cancer therapies. The findings from this study could pave the way for more effective treatment options, ultimately improving patient outcomes and quality of life. Thus, this research aims to explore the potential of magnetic nanoparticles in cancer therapy and address the pressing need for safer and more effective treatment modalities in the fight against cancer.

## 2. Materials and Methodology Materials

### 2.1. PBS Synthesis and TDI Modification

The 1.4 butanediol P.S. (99.3%), the succinic acid P.A. ACS (99.0%), the orthophosphoric acid P.A. (99.0%), the silicone grease (99.0%), and the chloroform P.A. (99.8%) were purchased from VETEC (Duque de Caxias, Brazil), while the toluene diisocyanate (95%) was obtained from Reduc (Duque de Caxias, Brazil).

### 2.2. PBS-PEG Synthesis

For the PBS-PEG synthesis, we used PBS modified with TDI. The orthophosphoric acid P.A. (99.0%) and polyethylene glycol 5000 (98.5%) were purchased from VETEC (Duque de Caxias, Brazil) and Sigma Aldrich (São Paulo, Brazil), respectively.

### 2.3. PBS-PEG Purification

For PBS-PEG purification, we purchased the ethyl alcohol and dichloromethane from Synth (Diadema, Brazil) and VETEC (Duque de Caxias, Brazil).

### 2.4. Magnetite Synthesis

For the magnetite synthesis, iron chloride III, hexahydrate, potassium hydroxide, ferrous sulfate, and sodium citrate were purchased from VETEC (Duque de Caxias, Brazil), while 200 nm porous filters were obtained from Chromafil (Düren, Nordrhein-Westfalen, Germany).

### 2.5. Nanoparticle Synthesis

For the nanoparticle synthesis, we used the dichloromethane purchased from VETEC (Duque de Caxias, Brazil), and PBS-PEG, magnetite, and polyvinyl alcohol (PVA) were purchased from Sigma Aldrich (São Paulo, Brazil).

## 3. Methods

### 3.1. PBS Synthesis

PBS synthesis consumed 35.9 g of succinic acid, 27 mL of 1,4-butanediol, and 0.1 mL of phosphoric acid, which is the catalyst. These reactants were mixed in a three-necked flask, which was closed under nitrogen flow. A slight vacuum was applied using a pump. A balloon into an ice bath was used at the condenser outlet, reducing the water temperature and preventing boiling. The trap was continuously cooled using liquid nitrogen. The system temperature was equal to 150 °C, and the system was under magnetic stirring for 7 h.

### 3.2. PBS Modification with TDI

PBS modification with TDI (Toluene Diisocyanate) was performed using 10 mg of PBS in 50 mL of chloroform and kept stirring for 15 min. Then, 1 mL of TDI was dropped inside the system, which was kept while stirring stirring for another 10 min.

### 3.3. PBS-PEG Synthesis

PBS-PEG synthesis consumed 0.05 mL of phosphoric acid, which was added to the PBS solution containing TDI, and 10 mg of PEG, solubilized in 50 mL of water in a separate beaker. Finally, the solutions were transferred to a single beaker, producing a biphasic mixture, and stirring was continued for 15 min. Then, the mixture was left quiescent for 24 h, producing a PBS-PEG film at the interface between the two phases. The PBS-PEG purification was performed by dissolving it in dichloromethane. After dissolution, the precipitation was performed using ethanol in excess. After 24 h, a process of vacuum filtration, washing, and drying at room temperature of 26.5 °C for 48 h was performed.

### 3.4. Magnetite Nanoparticle Synthesis

Magnetite nanoparticle synthesis consumed 25 mL of an aqueous solution of ferric chloride hexahydrate (2.5 mmol) and 25 mL of ferrous sulfate heptahydrate (2.5 mmoles). The two solutions were mixed in a beaker, where an additional 25 mL aliquot of deionized water was added and left under stirring for 15 min. At the same time, 25 mL of a potassium hydroxide solution (2 moles) was prepared; 22.5 mL of that solution was added to the complex under stirring. The stirring conditions were kept for another 30 min. Then, the washing was performed. At this point, a magnet was placed below the beaker, making the supernatant remotion accessible until the neutral pH. The filtration step was performed using a filter (pores of 200 nm) coupled with a syringe. Finally, the magnetite nanoparticles were lyophilized.

### 3.5. Magnetite Nanoparticle Encapsulation

The nanoparticle modification followed the emulsion/evaporation method. The emulsion contained an aqueous phase filled with polyvinyl alcohol (PVA—0.5% *w*/*w*). The organic phase used dichloromethane as the solvent, where PBS-PEG (1% *w*/*w*) was dissolved and magnetite nanoparticles (0.3% *w*/*w*) were added. In a typical experiment, 3 mL of the organic phase was dropped on 30 mL of the aqueous PVA solution using a glass syringe coupled to a needle. The organic/aqueous mixture was homogenized for 15 min at a speed of 30,000 rpm through a Turrax homogenizer and a 100 mL beaker immersed in an ice bath. The obtained particles were washed with deionized water and centrifuged three times. The supernatant was discarded to eliminate the PVA excess present in the solution.

### 3.6. Dynamic Light Scattering (DLS) and Zeta Potential

The zeta potential and size distribution measurements of nanoparticles were performed by determining the electrophoretic mobility of the particles at 25 °C, using the Zetatrac equipment from (Microtrac, Montgomeryville, PA, USA), with a Teflon cell containing a pair of palladium electrodes. An aliquot of 1 mL of solution containing free or encapsulated magnetite nanoparticles was diluted with deionized water to a final volume of 5 mL and subsequently placed in the Teflon cell and analyzed, obtaining the zeta potential values and a graph of the size distribution of nanoparticles.

### 3.7. Scanning Electron Microscopy (SEM) and Energy Dispersive Spectroscopy (EDS)

SEM analyses were carried out with diluted solutions, where aliquots were obtained with a Paster pipette and dispersed onto a double-sided conductive film that was fixed to the sample holder. The samples were studied in a JEOL JSL 5300 Microscope (Jeol Instruments, Akishima, Tokyo, Japan). The encapsulated magnetite nanoparticle samples were coated with a thin layer of gold to prevent electrostatic charge accumulation on the material surface. Together with SEM, energy dispersive X-ray (EDX) was performed, which is an essential accessory in the microscopic characterization of materials. When an electron beam strikes a mineral, the outermost electrons of the atoms and constituent ions are excited and change energy levels. Upon returning to their initial position, they release the acquired energy, which is emitted at a wavelength in the X-ray spectrum. A detector installed in the vacuum chamber of SEM measures the energy associated with this electron. Since electrons of a particular atom have distinct energies, it is possible, at the point of incidence of the beam, to determine which chemical elements are present at that location and thus identify at an instant which mineral is being observed. The reduced diameter of the beam allows for the determination of mineral composition in very small samples, allowing for almost a pinpoint analysis. In this way, we can observe the differences in composition of magnetite nanoparticles before and after encapsulation.

### 3.8. X-Ray Diffraction (XRD)

The (Rigaku Miniflix X-ray diffractometer, Akishima, Tokyo, Japan) was used to analyze the magnetite nanoparticles. The equipment’s radiation source is 40 KV voltage and 20 mA current. The equipment’s target is a copper plate, producing CuKα radiation with a wavelength of 0.154 nm. The scan was performed at a 2θ angle, ranging from 25 to 70° at a fixed time (FT), with a step of 0.05 °/min.

### 3.9. Fourier Transform Infrared Spectroscopy (FTIR)

The analyses were performed on a (Perkin-Elmer 1720X Fourier transform spectrometer, Waltham, MA, USA), to which a diamond crystal ATR accessory was attached. The analyzed range was from 4000 to 400 cm^−1^, with a resolution of 4 cm^−1^ and under inert atmosphere.

### 3.10. Simultaneous Thermal Analysis (STA)

Term thermogravimetric analysis (TGA) and differential scanning calorimetry (DSC) were simultaneously performed using the (Perkin Elmer STA 6000, Waltham, MA, USA), equipment under a nitrogen atmosphere (purge gas—20 mL·min^−1^) and in the temperature range between 30 °C and 700 °C, with a heating rate of 20 °C/min. The samples were placed in an open alumina pan. The sample mass was adjusted to approximately 15 mg. Prior to the first analysis, an empty crucible (blank) run was performed to ensure equipment stability.

### 3.11. Magnetic Induction

Magnetic induction hyperthermia tests were performed using an Ambrell EASYHEAT L1 model. The solutions with encapsulated magnetite nanoparticles were placed in a beaker that was thermally isolated by a cellulose blanket. Soon after, each sample was inserted into the coil of the inductor. A factorial was made varying the time and the power to which the samples would be exposed, where the minimum factor (−) for the time and power were respectively 300 s and 450 A, the central factor (0) for the time and power were respectively 600 s and 600 A, and the maximum factor (+) for time and power were respectively 900 s and 750 A, as shown in Table 1, where each test was carried out in triplicate.

## 4. Results and Discussion

### 4.1. X-Ray Diffraction (XRD)

**Figure 1:**
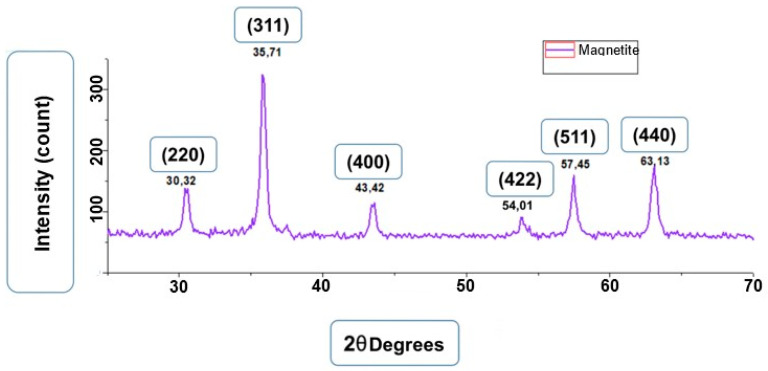
XRD spectrum of magnetite.

Figure 1 shows the diffractogram of the magnetite. In this figure, it was possible to identify the diffraction peaks at angle 2θ in 30.3°, 35.7°, 43.4°, 54.0°, 57.45°, and 63.1°. These peaks can be attributed to the Miller indices of (220), (311), (400), (422), (511), and (440), respectively.

The 2θ diffraction angles determined in the sample were similar to those presented in the standard diffractogram [17]. Thus, through this study, it was possible to confirm that the crystalline planes present in the sample produced correspond to the magnetite crystals.

The particle size was calculated according to Equation (1), using the diffraction peak that occurs at an angle of 2θ equal to 35.71°. The calculated average size was 18 nm. This is an indication that these particles may be superparamagnetic [9].

*Lc* = *K*λ/(β*Cos*θ) (1)

### 4.2. Fourier Transform Infrared Spectroscopy (FTIR)

**Figure 2:**
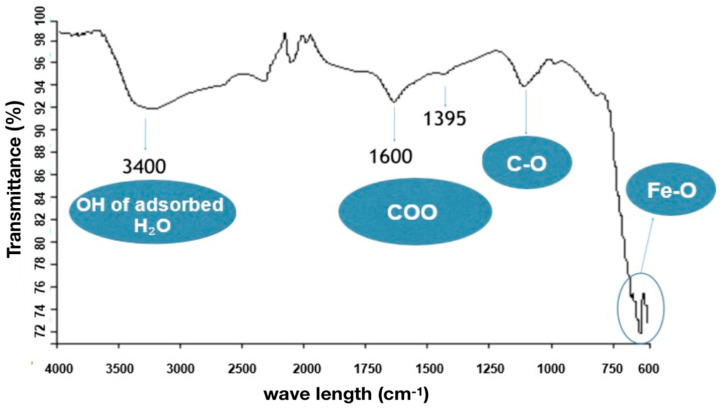
FTIR spectrum of magnetite.

Figure 2 shows the FTIR magnetite spectrum. A band of high intensity in the length of 630 cm^−1^, which can be attributed to the vibrations of the Fe-O bond of the iron oxide, was observed. The wideband, observed around 3400 cm^−1^, is typical of the stretching of hydroxyl groups (OH) present on the surface of the particles or due to residual water, common in magnetite from syntheses at low temperatures. It was also possible to observe peaks in 1600 cm^−1^ and 1395 cm^−1^, molecular vibrations that occurred in the COO- (carboxyl) group, and 1106 cm^−1^ vibrations that occurred in the C-O bond, coming from the sodium citrate. The appearance of these bands can confirm that the synthesis of magnetite has been successful.

**Figure 3:**
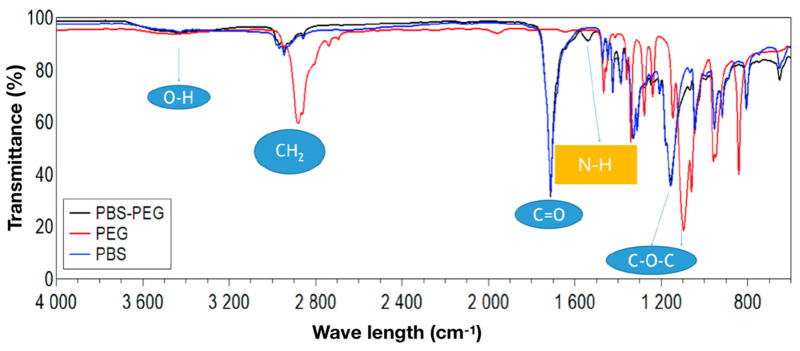
FTIR spectrum of PBS, PEG, and PBS-PEG polymers.

The analysis of the polymers was carried out well; the spectrum of Figure 3 shows the presence of the characteristic bands. The peaks at 2882 cm^−1^ and 2945 cm^−1^ are related to the vibrations of CH_2_ symmetrical and asymmetrical deformation. The peak, located at 1099 cm^−1^ and coming from the C-O-C group of an aliphatic ether, while the peak at 1157 cm^−1^ corresponds to the stretching of the C-O-C group coming from the aliphatic ester group.

**Figure 4:**
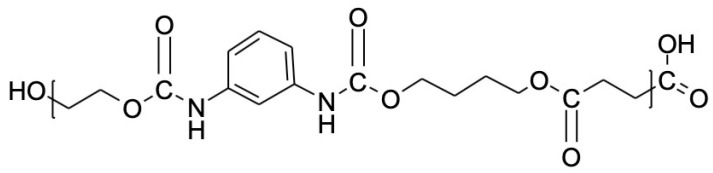
Structure of the PBS-PEG.

The peaks at 1711 cm^−1^ are related to the vibration of the C=O bond, which also comes from the ester group. Absorptions present around 3400 cm^−1^, corresponding to the stretching of the –OH bond. The 1539 cm^−1^ peak belongs to the NH bond present in the urethane bonds, which appears only in the PBS-PEG copolymer (Figure 4), indicating that the connection between the PBS and PEG polymers using TDI was successful, as well as the presence of the signed bands, which is a strong indication of obtaining the copolymer.

### 4.3. Thermogravimetric (Tga) and Differential Scanning Calorimetry (Dsc) Analyses

**Figure 5:**
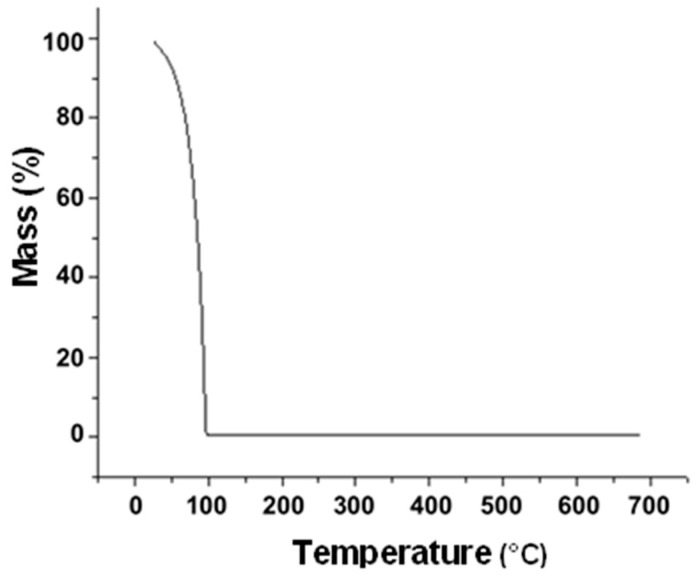
TGA graph of magnetite nanoparticles.

Figure 5 has the TGA graphic of magnetite in an aqueous environment after being filtered, so we can observe an abrupt drop in the mass that ends at 100 °C due to the mass loss of water. After that point, we no longer see mass loss, which was expected, since the magnetite does not suffer a significant mass loss in the experiment’s temperature range until 700 °C. A residual magnetite mass of 0.52% was obtained.

**Figure 6:**
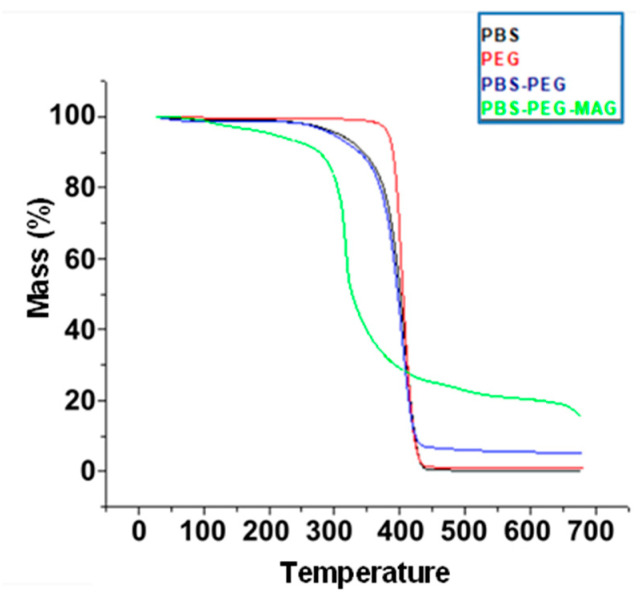
TGA graph of PBS, PEG, PBS-PEG polymers, and encapsulated magnetite nanoparticles.

Figure 6 shows the thermograms of the polymers: PBS, PEG, PBS-PEG, and the encapsulated magnetite. The degradation of PBS starts at 230 °C, like PBS-PEG. PEG begins to lose mass at 367 °C, while the magnetite encapsulated in PBS-PEG begins to lose mass at 135 °C. This result indicates that the magnetite generated a great loss of thermal stability in the polymer, probably due to some catalytic effect [18]. The highest rate of degradation of PBS was obtained at a temperature of 412.5 °C; in PEG, it was in 411 °C; in PBS-PEG, it was in 407 °C, while in encapsulated magnetite, it was in 319.5°C. PBS practically stops mass loss at 482 °C, while PEG loses mass up to 455 °C, PBS-PEG loses most of the mass up to 487 °C, and encapsulated magnetite up to 525 °C. The percentage of PBS residual mass at the end of the analysis was 0.136%, while PEG had a final value of 0.78% and PBS-PEG had a residual mass of 5.27%, probably due to some degree of crosslinking copolymer, generating a material that did not undergo depolymerization. In turn, the encapsulated magnetite generated a residue of 15.38%. A higher percentage of residual mass of the encapsulated magnetite was already expected, given the greater thermal stability of magnetite. With these values, using a simple interpolation, we can find an encapsulation efficiency of 36.59%.

**Figure 7:**
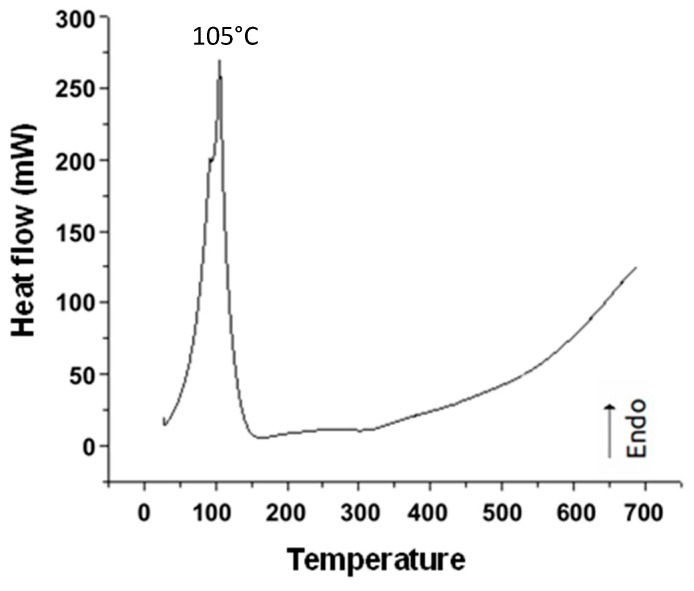
DSC graph of magnetite nanoparticles.

Figure 7 shows the DSC of magnetite in an aqueous environment, where we can observe the endothermic peak at 105 °C due to water evaporation.

**Figure 8:**
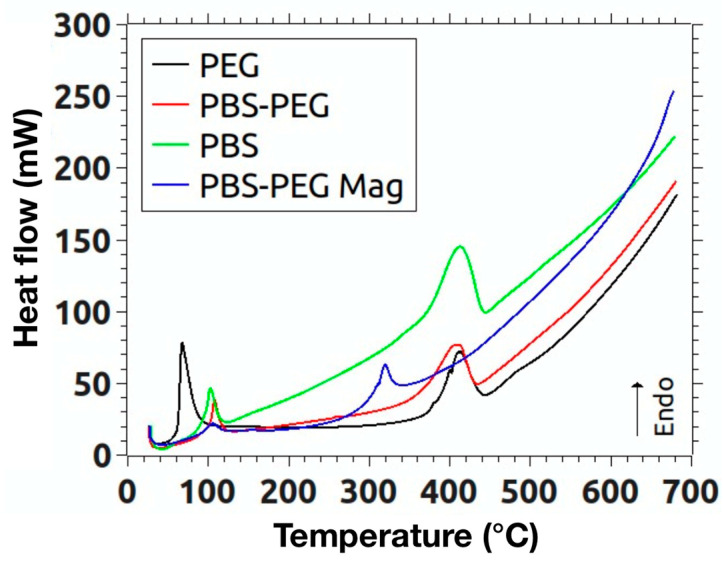
DSC graph of PBS, PEG, PBS-PEG polymers, and encapsulated magnetite nanoparticles.

DSC analyses of the polymers: PBS, PEG, PBS-PEG, and encapsulated magnetite nanoparticles were also performed (Figure 8). In this graph, we have the melting endotherms of the polymers and the encapsulated magnetite. It is clear that the first peaks refer to a phase change of the samples due to the absence of mass loss in the TGA curves in the temperature range in which the peaks occur. The PBS has a melting peak at 102.6 °C, while in the PEG the peak is located at 67.5 °C. The melting peak of PBS-PEG is at 107 °C and that of encapsulated magnetite at 105 °C. From the graph, it is also possible to obtain the enthalpy value of the phase change of each polymer and of the encapsulated magnetite. PBS presented a ΔHf value of 59.8 J/g, corresponding to the energy required for polymer fusion, while, in the PEG, the ΔHf value was 148 J/g and, in the PBS-PEG, it was 58.7 J/g. In turn, composite has ΔHf = 36.7 J/g. The second endothermic peak represents the degradation of polymers and encapsulated magnetite. In PBS, the second endothermic peak was observed at 412.5 °C with a ΔH value of 280.2 J/g. In PEG, the peak was observed at 410.7 °C with a ΔH value of 147.8 J/g. PBS-PEG has the second peak at 407 °C and a ΔH value of 262.2 J/g, while the encapsulated magnetite presented the second peak at 319.5 °C with a ΔH value of 57.2 J/g. We can see that PBS-PEG showed the first endothermic peak with a temperature higher than PEG and PBS, probably due to the fact that PBS crystals are surrounded by PEG chains, increasing the thermal gradient. So, we can see an increase in the melting temperature, but we do not see an increase in the enthalpy of PBS-PEG compared to PBS. The encapsulated magnetite curve showed a different profile from other polymers, with lower enthalpy values and lower degradation temperature, which corroborates the idea of some catalytic effect generated by nanoparticles.

### 4.4. Dynamic Light Scattering (DLS)

**Figure 9:**
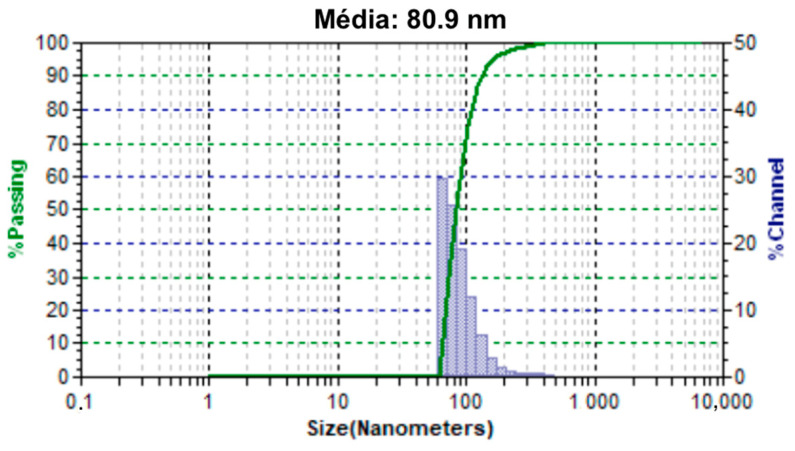
Size distribution of the magnetite nanoparticles obtained by (DLS).

In the DLS analysis, we can observe the size distribution of the magnetite nanoparticles (Figure 9). An average size of 80.9 nm of the magnetite nanoparticles was obtained; probabilistic aggregates of about 91 magnetite nanoparticles since the analysis of XRD allowed the calculation of a size of 18 nm for the nanoparticles. The graph also shows that the filtering of particles using a 200 nm pore filter was efficient, as the percentage of particles with sizes greater than 200 nm was insignificant.

**Figure 10:**
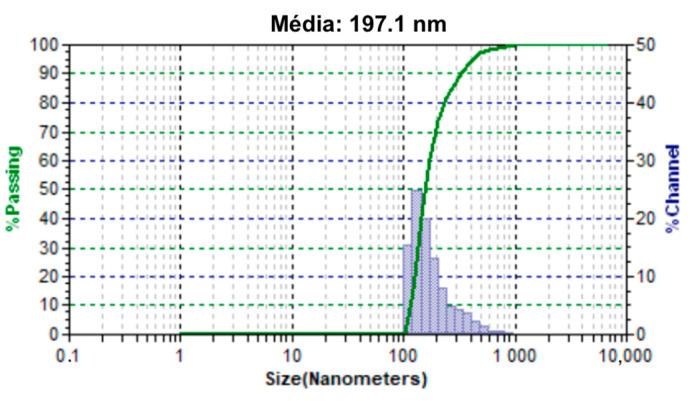
Size distribution of the encapsulated magnetite nanoparticles obtained by DLS.

Another DLS analysis was performed with the magnetite nanoparticles encapsulated with PBS-PEG. We can see, in Figure 10, the size distribution of the nanoparticles, where the average size was 197.1 nm and 70% of the nanoparticles are concentrated in the 100–200 nm range. This is a good feature for particles that will circulate in the bloodstream. These particles, when administered intravenously, may preferentially accumulate in tumors. This accumulation is due to the permeability and retention effects. Particles with a size of around 200 nm passively enter tumor blood vessels [19]. Circulating macrophages and reticuloendothelial cells residing in the spleen and liver recognize these particles as foreign and surround and eliminate them from the circulation. However, particles sized at 200 nm can escape detection by the immune system, resulting in prolonged circulation times for these nanoparticles. This increases the circulation time of these nanoparticles [20]. Another important fact that we can observe is that we do not have particles larger than 1000 nm, which makes this formulation able to be administered intravenously without risks to the user since the diameter of the smallest capillary vessels is around 5 to 6 μm, and so that embolism does not occur, the administered particles must be significantly smaller [21]. Through this analysis, we can make another important observation: after the magnetite nanoparticles were encapsulated, we did not observe particle sizes smaller than 100 nm. Where the largest portion of magnetite nanoparticles aggregates were found, the size distribution graph shifted, causing most of the particles to be in the range of 100–200 nm, which shows an increase in size generated by the PBS-PEG layer around the nanoparticles of magnetite. This increase in particle size due to the formation of polymer layers on the particle surface was also observed in the work [22].

### 4.5. Zeta Potential

The stability of a suspension depends on the physical properties of the colloidal particles that constitute it, and it is necessary to determine them to understand the individual interactions of each particle that can lead to the destabilization of a suspension. In general, we seek to maximize the repulsive forces between the particles in order to minimize the interactions that lead to the formation of aggregates, which destabilize colloidal suspensions. Thus, the interaction forces on the colloid surfaces are responsible for the behavior of the suspensions, with repulsive electrostatic forces being one of the ways through which the particles can resist aggregation. Potentials in the range between ±40 and ±60 guarantee good colloidal stability [23]. Hence, measurements of the zeta potential were obtained from the nanoparticles in order to predict the aggregation trend.

The analysis of zeta potential demonstrated, as we can see in Table 2, that the nanoparticles of magnetite have small surface zeta potential. Consequently, it has a strong tendency to cluster, a fact that we can observe with the analysis of DLS (Figure 9), where the smallest nanoparticle size obtained was between 60 nm and 70 nm, while the DRX indicated that the size of magnetite nanoparticles was 18.1 nm, thus evidencing the strong tendency of these nanoparticles to agglomerate, while encapsulated magnetite nanoparticles have a higher surface charge, which decreases the tendency to agglomerate because the greater the zeta potential, the greater the repulsion between the particles. This result shows that the encapsulation of magnetite with PBS-PEG improved an important property for a colloidal system, ensuring greater stability and less tendency for aggregation between the particles.

### 4.6. Scanning Electron Microscope (SEM) and Dispersive Energy Spectroscopy (EDS)

**Figure 11:**
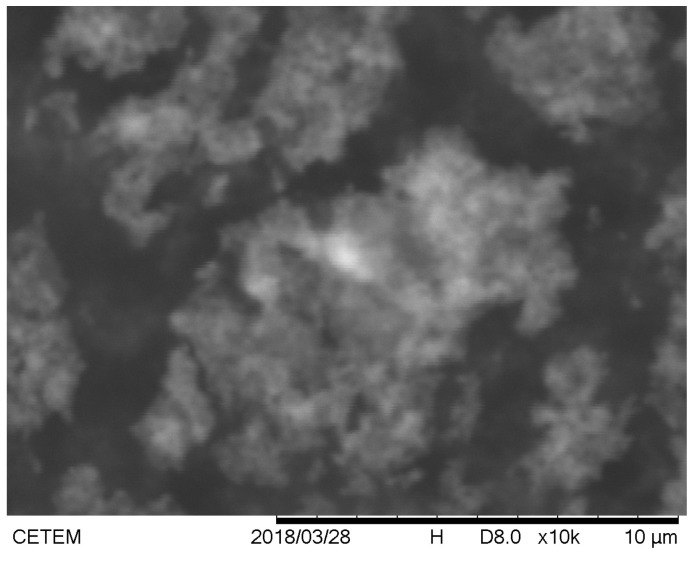
SEM image of magnetite nanoparticles.

Images of the magnetite nanoparticles were made in the SEM, where we can see that the particle size is indeed a nanometric size because we worked at the maximum resolution of the SEM. Still, it was not enough to clearly visualize the magnetite nanoparticles. However, we can observe the regions where nanoparticles are found, which appear in Figure 11 as lighter regions.

**Figure 12:**
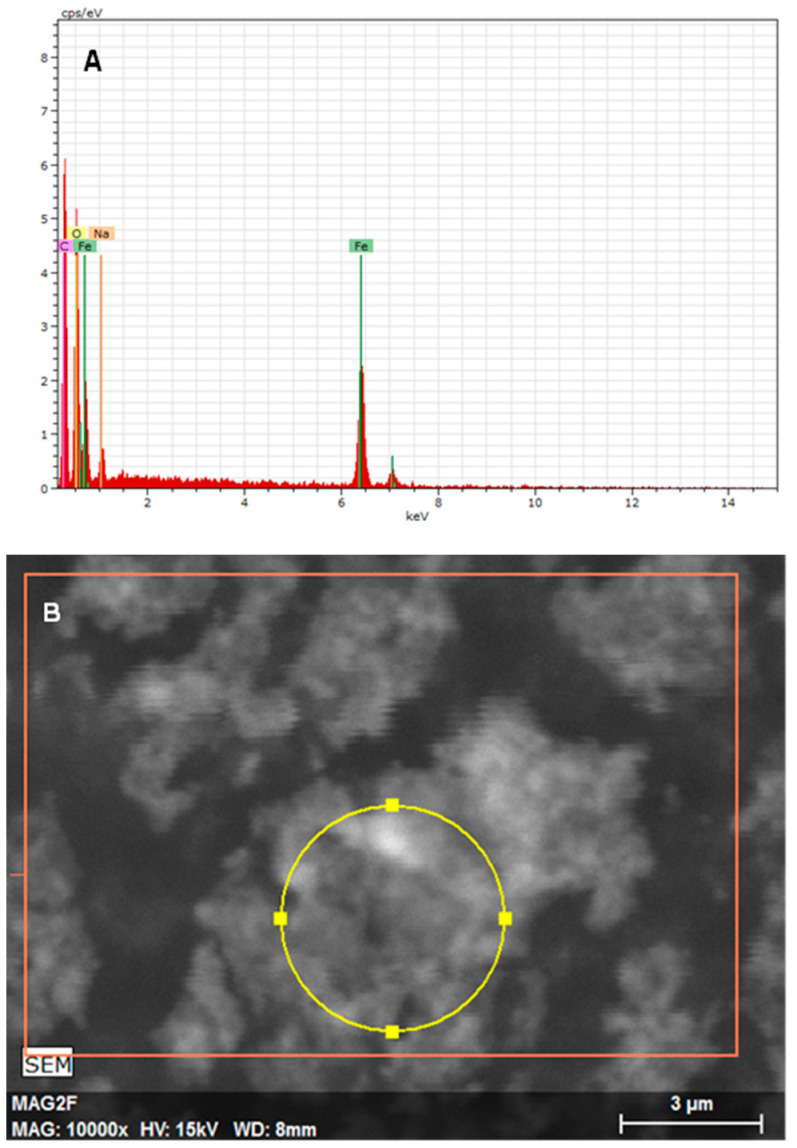
(**A**) Graph of the EDS spectroscopy of the magnetite nanoparticles, (**B**) image obtained in the SEM of the region analyzed by the EDS.

From the analysis of the EDS, we can confirm that the lighter region really is the nanoparticles of magnetite, with the peaks that characterize the iron (Fe) and oxygen (O) appearing in the analysis with prominence, ensuring that, in the region marked with the yellow circle in Figure 12A, there is a good concentration of magnetite (Fe_3_O_4_).

**Figure 13:**
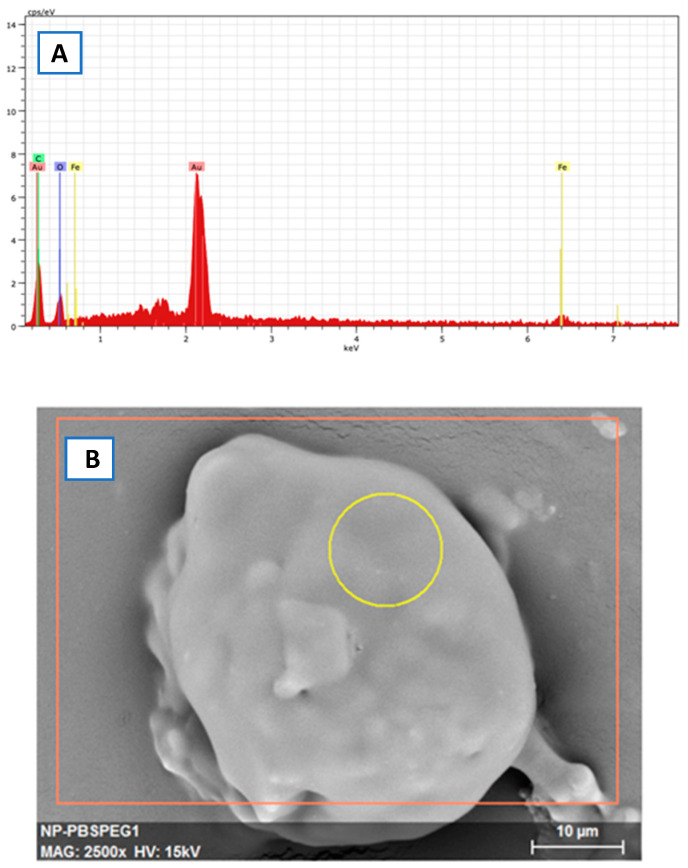
(**A**) Image obtained, in the SEM, of the region analyzed by the EDS, (**B**) graph of the EDS spectroscopy of the encapsulated magnetite nanoparticles.

In the analysis of the EDS, we can see, in Figure 13, that the analyzed region marked with a yellow circle in image B has peaks in the regions of carbon (C) and oxygen (O), which are elements present in PBS-PEG. We can also observe a smaller peak and more extension of iron (Fe) compared to EDS performed with free magnetite nanoparticles; this is due to the fact that the magnetite is covered with PBS-PEG, which makes the presence of Fe less noticeable for the equipment. The gold peak (Au) appears on the graph because the sample was covered with gold to improve the resolution of the images.

### 4.7. Magnetic Induction

We can see in Table 3 the results of the factorial design in relation to the temperature variation. The concentration used for the tests was 4.217 mg/mL of nanoparticles. Using the shortest time (−) and lowest current (−) of the planning, an average of △T = (1.47 ± 0.25) °C was obtained, while by using the maximum time (+) and maintaining the lowest current (−), we obtained a greater variation in temperature as expected, with an average of △T = (2.67 ± 0.15) °C. And, when using the shortest time (−) and the highest current (+) of the factorial, a mean of △T = (2.43 ± 0.16) °C was obtained, which was very close to the variation of tests 4, 5, and 6. The results for the midpoint of the factorial, with an intermediate variation of time (0) and current (0) and average of △T = (2.2 ± 0.26) °C, were obtained. In the samples with the longest time (+) and highest current (+), an average of △T = (4 ± 0.27) °C was obtained, with the highest △T of the planning.

**Figure 14:**
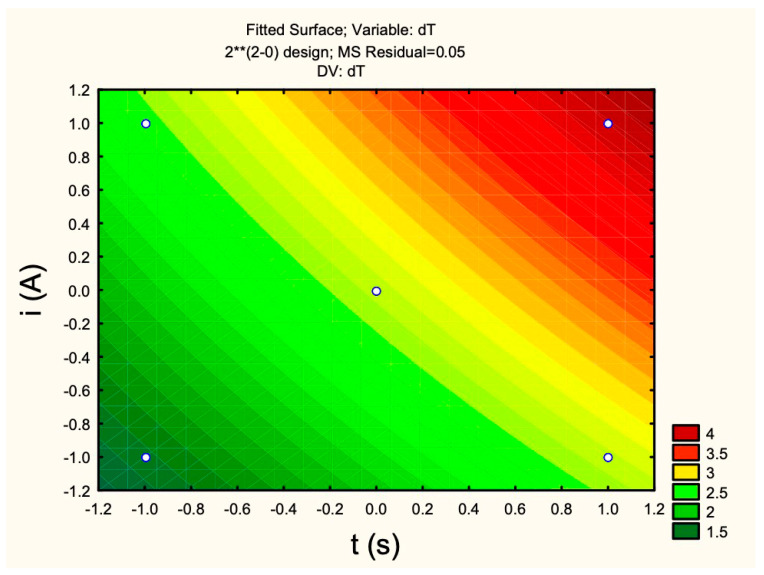
Current versus time statistical study of hyperthermia tests for encapsulated magnetite nanoparticles. 2** = Two factors at two levels plus a central point.

In order to better understand these results and observe the variables that influence the temperature increase, a statistical analysis was carried out in the STATISTICS program, with 95% confidence, which is shown in Table 4. Table 4 and Figure 14 show the statistical study carried out for encapsulated magnetite nanoparticles, and according to this, current (2)i had a positive influence on temperature, with an effect of magnitude 1.15 and a standard error of 0.13 with a degree of distrust (*p*) of 0.000005. Time (1)t also showed a positive influence with an effect of magnitude 1.38 with a standard error of 0.13 and a degree of distrust (*p*) of 0.000001. The central point allows for analyzing the curvature on the response surface, indicating the possible presence of a local minimum or maximum. The synergy between current and time did not have a significant effect on temperature either.

Figure 14 shows the effect of current and time on the temperature, where it is clear that, when we increase the current or time, we have an increase in temperature.

**Figure 15:**
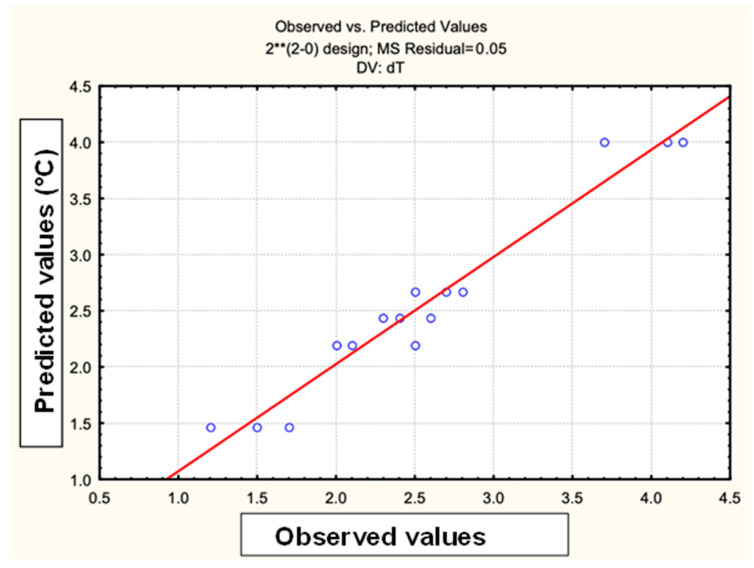
Statistical study predicted values versus observed values of the hyperthermia test for encapsulated magnetite nanoparticles. 2** = Two factors at two levels plus a central point.

Figure 15 has a graph of predicted values versus observed values. Based on the results obtained, the STATISTICA program proposes a model predicting what the temperature value will be for each point of the experiment, which is represented by the red line and the values obtained by the blue circles.

In this context, a quantity of fundamental importance is the release of heat normalized by a mass of magnetic particles, called SAR (specific absorption rate). Considering the dispersion of magnetic particles in water and assuming that the total specific heat of the system, weighted by the mass of the system, is equivalent to the sum of the specific heats weighted by the respective masses of magnetic particles and water alone, we can use Equation (2) to calculate the SAR, where (C_np_) is the specific heat of the particle, (C_H2O_) is the specific heat of water, (ρ_H2O_) is the specific mass of water, (ρ_np_) is the concentration of particles, and ΔT/Δt is the heat release rate of the system.

SAR = [C_np_ + (ρ_H2O_ × C_H2O_/ρ_np_)] × ΔT/Δt (2)

The hyperthermia tests performed in this study generated a SAR = 10,372 W/Kg, which is an excellent result. This can be seen in the study [22], which obtained a SAR = 3762 W/Kg, a value, according to the author, more than enough to kill cancer cells using hyperthermia. With this result, we can see that nanoparticles have enormous potential in the application of hyperthermia for the treatment of cancer.

## 5. Conclusions

Based on the results obtained in this work, we can conclude that the PBS and PBS-PEG synthesis was successful, since the FTIR analyses indicate the presence of peaks related to the functions found in the PBS and the copolymer, as well as the urethane bonds. In the DSC analysis, we can observe the endothermic peak characteristic of the PBS fusion, as well as an enthalpy of intermediate degradation of PBS-PEG between the curve of PBS and PEG and the interference of magnetite nanoparticles in the polymer degradation temperature. The TGA analysis demonstrated something expected, which is the increase in the sample’s residual mass with the encapsulated magnetite if compared to the sample only with the polymer, proving that the magnetite was successfully encapsulated by PBS-PEG.

Based on the results obtained in FTIR, EDS, and DRX, the synthesis of magnetite nanoparticles was successfully performed. In the FTIR analysis, we can observe a characteristic magnetite peak referring to the Fe-O bond and peaks indicating the presence of sodium citrate. In the EDS, we can also observe peaks indicating the presence of Fe and O. In the XRD graph, we observed peaks at specific angles of the magnetite, and with that, it was possible to measure the size of the magnetite nanoparticles that presented a crystalline size of 18.1 nm. The size analysis of the magnetite nanoparticles using the DLS showed an average magnetite nanoparticle size of 80.9 nm, which indicates that the magnetite nanoparticles underwent aggregation. The encapsulated magnetite nanoparticles showed satisfactory results. The TGA and DSC analyses demonstrated that the presence of the magnetite nanoparticles in the copolymer generated thermal instability, changing the degradation temperature as well as its degradation enthalpy. In the SEM images, we can observe significant morphological differences between the free and encapsulated magnetite nanoparticles. In the EDS analysis, it was possible to see peaks referring to Fe, C, and O on the surface of the encapsulated magnetite nanoparticles, being another indication that the magnetite was encapsulated. Another important fact was seen in the DLS analyses: the difference between the average size of the free and encapsulated magnetite nanoparticles, where the encapsulated magnetite nanoparticles had a larger average size than the free magnetite nanoparticles, pointing out that the PBS-PEG covered the magnetite, increasing the diameter of the particles. The results also demonstrated that the nanoparticles can be administered intravenously, since no particles larger than 1000 nm were observed and the diameter of the smallest capillary vessels is around 5 to 6 μm.

In the analysis of zeta potential, we can see that the encapsulation of magnetite generated good results since it increased the zeta potential of the surface of the particles, which increases the repulsion between them and decreases the tendency of aggregation of the particles.

The magnetic induction tests demonstrated, through the factorial, that the current and the time were significant for the increase in the sample temperature. The tests also demonstrated that the nanoparticles presented a SAR = 10,372 W/Kg, which according to the literature, produces enough heat to generate the cancer cell’s death, which demonstrates the enormous potential of these nanoparticles in the use of hyperthermia in the treatment of cancer.

In vivo studies will be needed to evaluate nanocomposites’ therapeutic efficacy and safety in animal models before human trials. Further study of nanocomposites’ modes of action will reveal their effects on cancer cells and the immune system. This research provides a solid framework for future studies to improve and validate the cancer therapy method.

## Figures and Tables

**Table 1:** Factors and levels applied in the induction tests of encapsulated magnetite nanoparticles.

			Levels	
Factors Studied	−		0	+
Power (A)	450		600	750
Time (S)	300		600	900
**Samples**		**Power**		**Time**
1		−		−
2		−		+
3		+		−
4		+		+
5		0		0

**Table 2:** Zeta potential tests.

Samples	Zeta Potencial		
Free magnetite	2.51	2.79	7.13
Encapsulated magnetite	−53.35	−57.23	−51.64

**Table 3:** Temperature variation results obtained through the factorial.

Samples	Time	Power	Δt (°C)
1	−	−	1.2
2	−	−	1.7
3	−	−	1.5
4	+	−	2.8
5	+	−	2.7
6	+	−	2.5
7	−	+	2.3
8	−	+	2.4
9	−	+	2.6
10	+	+	3.7
11	+	+	4.2
12	+	+	4.1
13	0	0	2.0
14	0	0	2.1
15	0	0	2.5

**Table 4:** Statistical study of hyperthermia tests for encapsulated magnetite nanoparticles.

	Effects	Standard Error	*p*
Average/interaction	2.641667	0.064550	0.000000
Curvatr.	−0.883333	0.288675	0.012045
(1)t	1.383333	0.129099	0.000001
(2)i	1.150000	0.129099	0.000005
(1)t (2)i	0.183333	0.129099	0.185997

## Data Availability

The raw data supporting the conclusions of this article will be made available by the authors on request due to privacy.
